# MTFR2 Promotes the Proliferation, Migration, and Invasion of Oral Squamous Carcinoma by Switching OXPHOS to Glycolysis

**DOI:** 10.3389/fonc.2020.00858

**Published:** 2020-05-27

**Authors:** Wei Wang, Meihua Xiong, Lin Jiang, Zean Chen, Yisen Shao

**Affiliations:** ^1^Department of Oral and Maxillofacial Surgery, Affliated Hospital of Jiangxi University of Traditional Chinese Medicine, Nanchang, China; ^2^Department of ENT, Jiangxi Province of Integrated Chinese and Western Medicine, Nanchang, China

**Keywords:** oral squamous carcinoma, MTFR2, EMT, glycolysis, OXPHOS

## Abstract

MTFR2 is an oncogene involved in the progression of cancer, its' potential mechanism in oral squamous carcinoma remains unknown. The aim of this study was to uncover the bio-function and the mechanism of MTFR2 in the progression of oral squamous carcinoma. We scanned TCGA database to identify MTFR2 as dysregulated genes. qRT-PCR and Western blotting assays were applied to detect the expression pattern of MTFR2 in oral squamous carcinoma. We next established stable MTFR2-overexpressing and MTFR2 knocking down cell lines. A series of experiments were applied and the results indicated that MTFR2 was upregulated in cancer tissue and negatively correlated with the overall survival (OS) of patients in both the TCGA database and our inhouse database. Following experiments showed that MTFR2 promotes proliferation, migration and invasion in an oral squamous carcinoma cell line by switching OXPHOS to glycolysis.

## Introduction

Oral squamous cell carcinoma (OSCC) is the most common type of cancer in the head and neck area. It is one of the ten most common cancers and is listed as the most common malignancy in the Department of Oral and Maxillofacial Surgery (1). Patients still suffer from metastasis and recurrence when diagnosed at advanced stages, and no targeted therapy has been suitably applied in clinical trials. Metabolic imbalance has been detected in many kinds of cancers, and therapies targeting metabolism are an emerging field worldwide (2–4). Therefore, efforts are still in urgent need to develop effective metabolism-targeted therapies for OSCC.

Glycolysis is one of the metabolic hallmarks of cancer cells. Cancer cells tend to degrade glucose to lactic acid even in an environment supplied with oxygen, which is known as glycolysis (5). Normal cells degrade glycose through oxidative phosphorylation (OXPHOS) and produce more ATPs. In this study, we searched the TCGA database and identified MTFR2 as one of the most dysregulated genes and applied qRT-PCR and Western blotting to uncover its expression pattern and biological function. We next detected the OCAR and ECAR and the markers of OXPHOS and glycolysis, this result revealed that MTFR2 promotes proliferation, migration and invasion in oral squamous carcinoma cell lines by switching OXPHOS to glycolysis.

## Materials and Methods

### Tissue Samples

All human tissues were obtained from the surgical suite in the Department of Oral and Maxillofacial Surgery, Affiliated Hospital of Jiangxi University of Traditional Chinese Medicine after confirmation by a pathologist. Tissues were obtained with the patients' written consent under a protocol approved by the institution's Institutional Review Board. The characteristics of patients were shown in Table S1.

### Cell Culture

HaCaT and CAL 27 were cultured in DMEM (Sigma) supplied with 10% fetal bovine serum (Invitrogen) and 1% penicillin–streptomycin while other cell lines were cultured in DMEM F-12 in the incubator. When treated with WZB117, cells were incubated in the complete medium supplied with 5 μg/ml of WZB117 for 24 h.

### Real-Time (RT) qPCR

Total RNA from cells or tissues was extracted using TRIzol reagent (Thermo Fisher Scientific). Quantitative RT PCR (qRT-PCR) was applied using SYBR Green PCR Master Mix (Takara Bio) on a CFX96 Real-Time PCR Detection System (Bio-Rad). Relative expression was calculated by normalization to β-actin. The primers for MTFR2 were designed automatically using the online tool Primer Bank (https://pga.mgh.harvard.edu/cgi-bin/primerbank), and the sequences are listed below:

forward primer: AGGGCTACGGGCCAATTTGA,

reverse primer: TTCCTAAATAAAGTTTGGTCCAC.

### Western Blot Assay

About 20 μg protein extract were separated by 12% SDS-PAGE. After transferring to a membrane and blocking, the corresponding primary antibodies were incubated overnight at 4°C. After incubation with the respective secondary antibodies for 1 h at room temperature. The protein bands were visualized using enhanced chemiluminescence reagents (Millipore).

### Colony Formation Assay

Two hundred cells per well were seeded and incubated for 2 weeks. The colonies were fixed using ice methanol for 10 min at room temperature and stained with 0.1% crystal violet for 20 min at room temperature.

### Trans-Well Assay

Migration and invasion were assessed using Trans-well plates following the manufacturer's protocol. A total of 5 × 10^4^ cells were resuspended in 250 μL of corresponding plain medium in the upper chamber (8-μm pore size, Corning,) while the lower chambers were filled with 750 μL of corresponding complete medium. After incubating for 24 h at incubator, the upper chambers were fixed with 100% ice methanol for 10 min and stained with 0.1% crystal violet at room temperature. The number of transmembrane cells was calculated under a microscope (Nikon,) at 200× magnification.

### Wound Healing Assay

Certain cells were seeded into the 6-well plate. After incubation for 24 h. The concentrate meets at 100%. Equal wounds were made by 1 ml pipette tips. The image of wound were obtained under a microscope (Nikon) at 100× magnification, the relative wound disclosure were calculated with the software ImageJ.

### Cell Counting Kit-8 (CCK-8) Assay

Five hundred cells per well were seeded into 96-well plates. The viability of cells was determined using a CCK-8 assay (Dojindo) everyday by measuring the absorbance at 450 nm (BioTek). The absorbance was normalized to the baseline.

### Statistical Analysis

Statistical analyses were performed using SPSS version 18.0 (SPSS Inc., Chicago, IL, USA). Data are presented as the mean ± SD. The chi-squared test was used to analyse clinicopathological characteristics. Student's *t*-test was applied to determine the difference in data. All experiments were repeated at least three times. A *P* < 0.05 was considered statistically significant.

## Results

### MTFR2 Is Upregulated in OSCC

**Figure 1:**
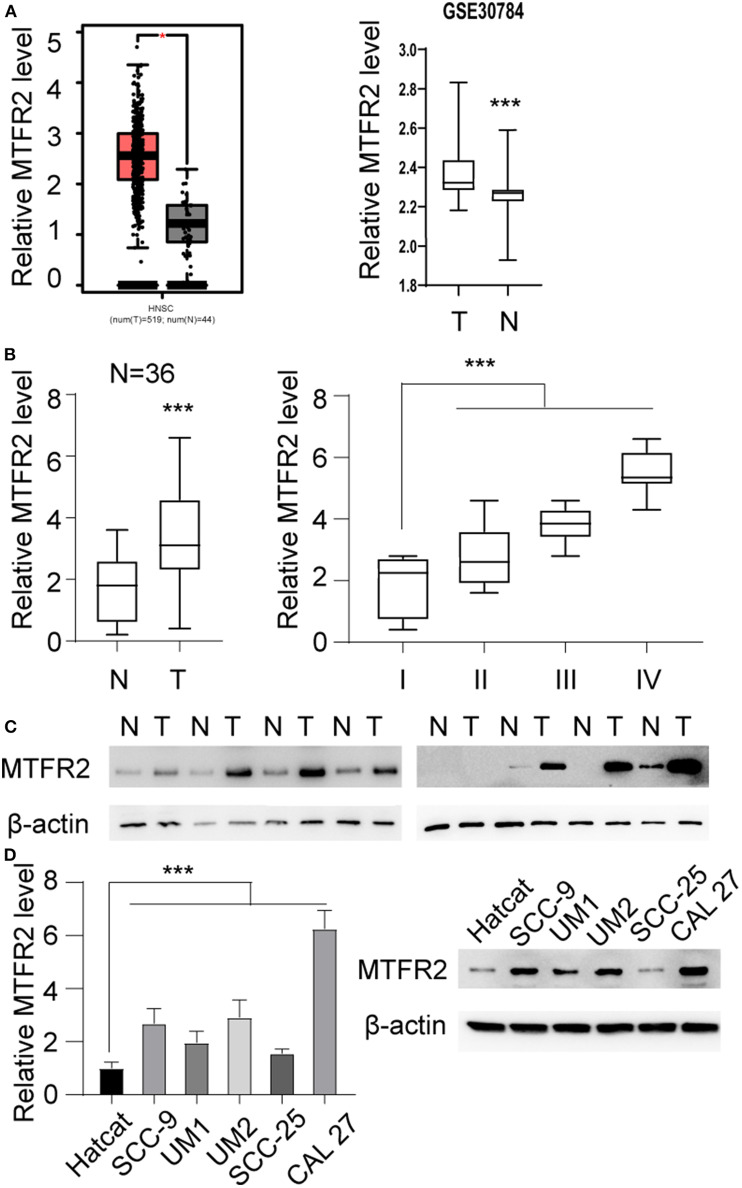
MTFR2 was upregulated in OSCC. **(A)** Left, the relative MTFR2 level in TCGA database, *p < 0.05 right, the relative MTFR2 level in GSE 30,784 database, ***p < 0.001. **(B)** Left, the relative MTFR2 level of in-house database, ***p < 0.001 right, the relative MTFR2 level in patients with different stage, ***p < 0.001. **(C)** The western blot of MTFR2 in 8 tumor and paired normal tissue. **(D)** Left, the relative MTFR2 level in OSCC cell line, ***p < 0.001. Right, the western blot of MTFR2 of OSCC cell line.

We scanned the TCGA database and identified that MTFR2 was upregulated in OSCC. The relative expression level is shown in Figure 1A, ^*^p < 0.05. MTFR2 was upregulated in 519 tumors compared with normal tissue. To further verify the expression pattern. We scanned the GEO databases and applied qRT-PCR in patients diagnosed with OSCC in our own department. The results are shown in Figures 1A,B. MTFR2 was upregulated in OSCC, and patients with higher levels of MTFR2 harbored high levels of MTFR2 (*p* < 0.001). We next detected MTFR2 in 8 paired tumor and normal tissues. MTFR2 was upregulated in 7 out of 8 tumors, while 1 tumor was undetectable (Figure 1C). We detected MTFR2 in OSCC cell lines at both the RNA and protein levels. MTFR2 was upregulated in the OSCC cell line compared with the normal HaCaT cell line, while Cal 27 harbored the highest level of MTFR2, and SCC-25 had the lowest level of MTFR2 (Figure 1D, *p* < 0.001).

### MTFR2 Promotes the Proliferation of OSCC

**Figure 2:**
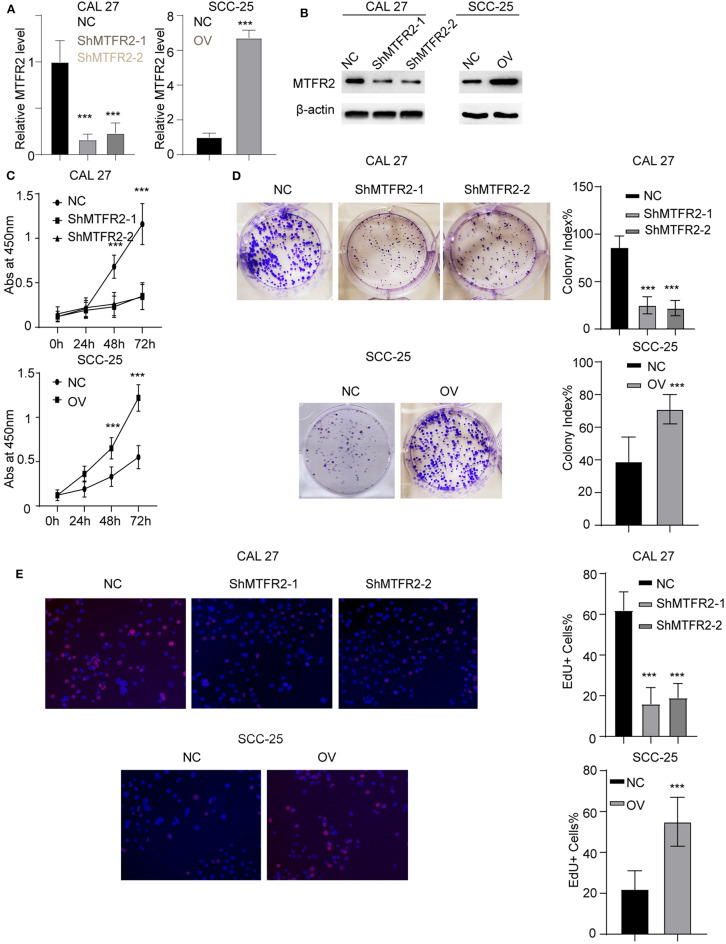
MTFR2 promotes the proliferation of OSCC. **(A)** The relative MTFR2 level in different cell lines. ***p < 0.001. **(B)** The western blot of MTFR2 in different cell lines. **(C)** The cck-8 assay in different cell lines. ***p < 0.001. **(D)** The colony formation assay in different cell line and statistical analysis, ***p < 0.001. **(E)** The EdU assay in different cell line and statistical analysis, ***p < 0.001.

We previously showed that MTFR2 was correlated with the progression of OSCC, To uncover the biological function of MTFR2 in OSCC, we next established stable cell lines knocking MTFR2 down and overexpression MTFR2. The RNA and protein levels in these cells are detected in Figure 2A, *p* < 0.001 (Figure 2B). We next applied the CCK-8 assay, colony formation assay and EdU assay. Cells with higher levels of MTFR2 harbor improved proliferation ability in the CCK-8 assay and the colony formation assay (Figures 2C,D, *p* < 0.001). MTFR2-overexpressing cells harbor higher percentage of EdU-positive cells (Figure 2E, *p* < 0.001). Taken above, MTFR2 promotes the proliferation of OSCC over both short time and long time periods.

### MTFR2 Promotes the Migration and Invasion of OSCC

**Figure 3:**
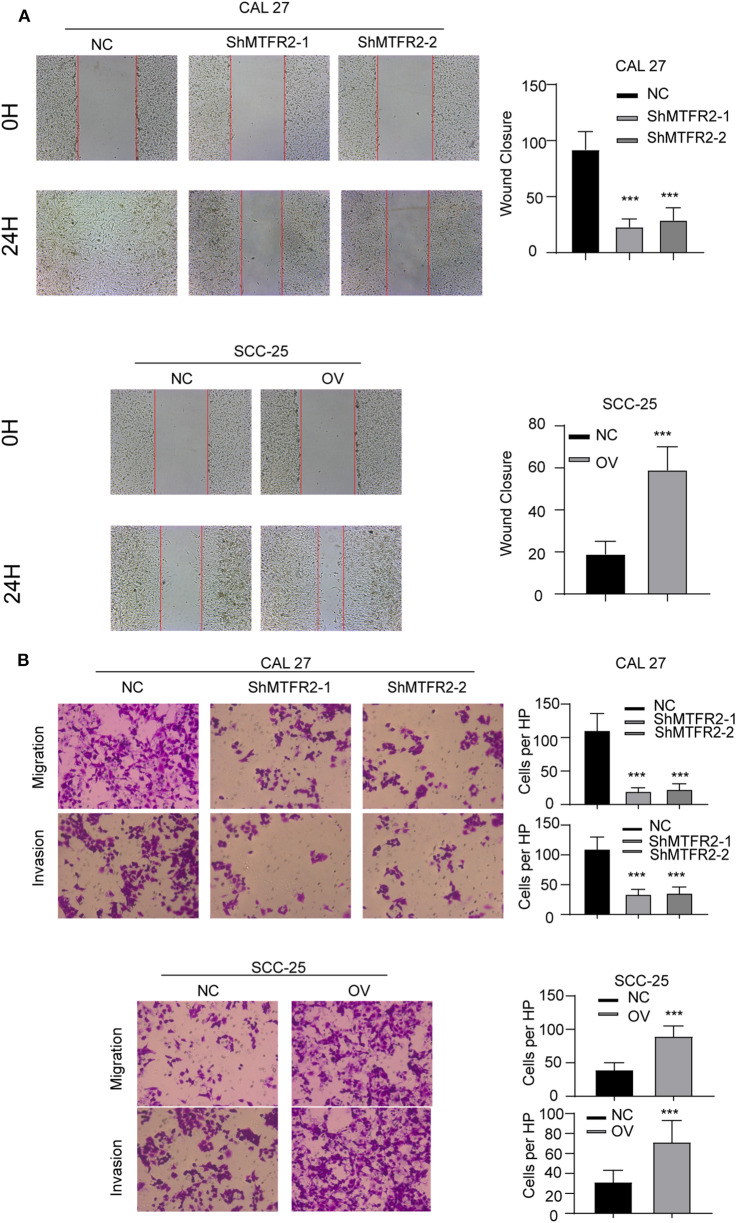
MTFR2 promotes the migration and invasion of OSCC. **(A)** The representative image of wound healing and statistical analysis, ***p < 0.001. **(B)** The representative image of trans-well and invasion chamber and statistical analysis, ***p < 0.001.

MTFR2 was upregulated in OSCC as previously described. We next examined the effect of MTFR2 in migration and invasion of OSCC cell lines. We applied the wound healing assay, the Trans-well assay and the invasion chamber assay as described above. The cells with higher levels of MTFR2 developed improved migration and invasion ability in wound healing (Figure 3A, *p* < 0.001) and Transwell and invasion chamber assays (Figure 3B, *p* < 0.001). We next applied glycolysis inhibitor in SCC-25 OV (Rescue), the migration and invasion ability totally restored (Figures S1A,B, ^***^p < 0.001). Taken together, MTFR2 promotes the migration and invasion of OSCC.

### MTFR2 Promotes the EMT in OSCC

**Figure 4:**
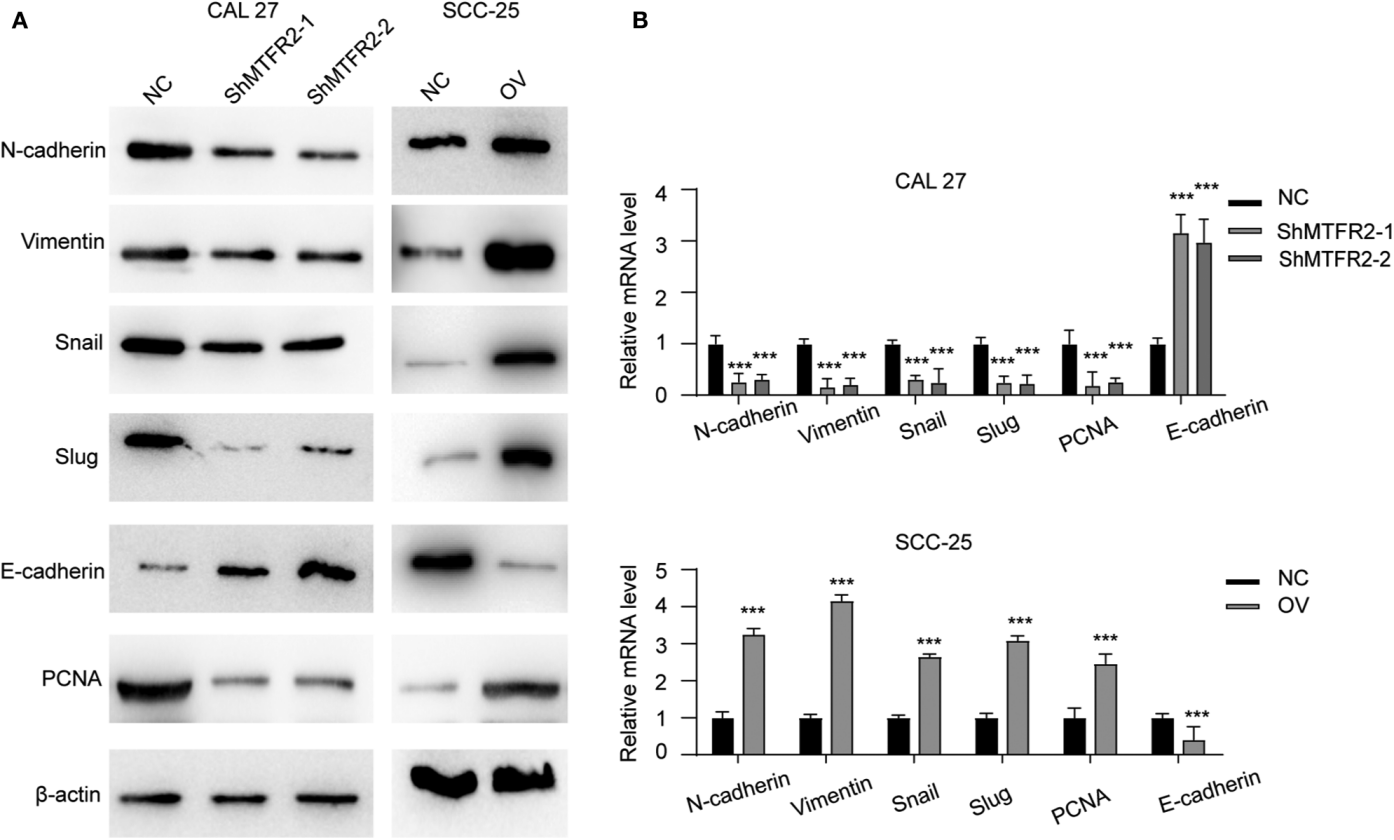
MTFR2 promotes the EMT of OSCC. **(A)** The western blot of EMT markers and PCNA in different cell line. **(B)** The relative mRNA level of EMT markers and PCNA in different cell line, ***p < 0.001.

MTFR2 promotes proliferation, migration and invasion in OSCC as previously described. We detected the expression pattern of EMT markers and PCNA in cell lines which reflect the mesenchymal status and proliferation status. Mesenchymal markers such as N-cadherin, snail and vimentin decreased with the knocking down of MTFR2, but epithelial markers such as E-cadherin increased in the shMTFR2 cell line; however, mesenchymal markers increased but epithelial markers decreased at both the RNA and protein levels in the MTFR2-overexpressing cell line compared to the control cell line (Figures 4A,B, *p* < 0.001).

### MTFR2 Regulates Glucose Metabolism by Switching OXPHOS to Glycolysis

**Figure 5:**
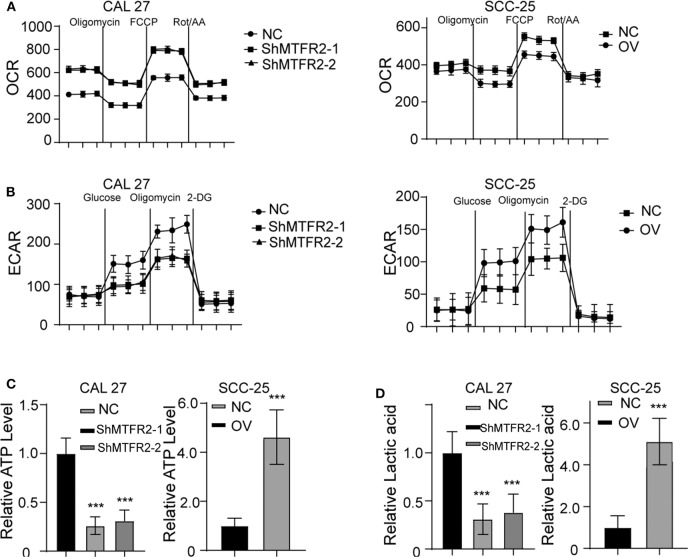
MTFR2 promotes the glycolysis of OSCC. **(A)** The OCAR assay in different cell line. **(B)** The ECAR assay in different cell line. **(C)** The relative ATP level in different cell line, ***p < 0.001. **(D)** The relative lactic acid level in different cell line, ***p < 0.001.

The potential function of MTFR2 in normal cells or cancer cells has rarely been studied. MTFR2 was predicted to be located in the mitochondria in the online predicting tool GeneCards (https://www.genecards.org/). Mitochondria are the most important organelles in glucose metabolism. We thus detected the OXPHOS and glycolysis status in the cell lines. The results are shown in Figures 5A,B. The OXPHOS rate decreased in MTFR2-overexpressing cells, while glycolysis increased in the cells with higher levels of MTFR2 compared to control cells. We hypothesized that glycolysis was enhanced, and we next detected ATP levels and lactic acid levels. Glycolysis efficiently produces ATP and lactic acid. The ATP and lactic acid levels increased in Cal 27-NC and SCC-25-OV cells compared to control cells (Figures 5C,D, *p* < 0.001). Taken together, we concluded that MTFR2 regulated glucose metabolism by switching OXPHOS to glycolysis.

### MTFR2 Promotes Glycolysis Metabolism Cascades

**Figure 6:**
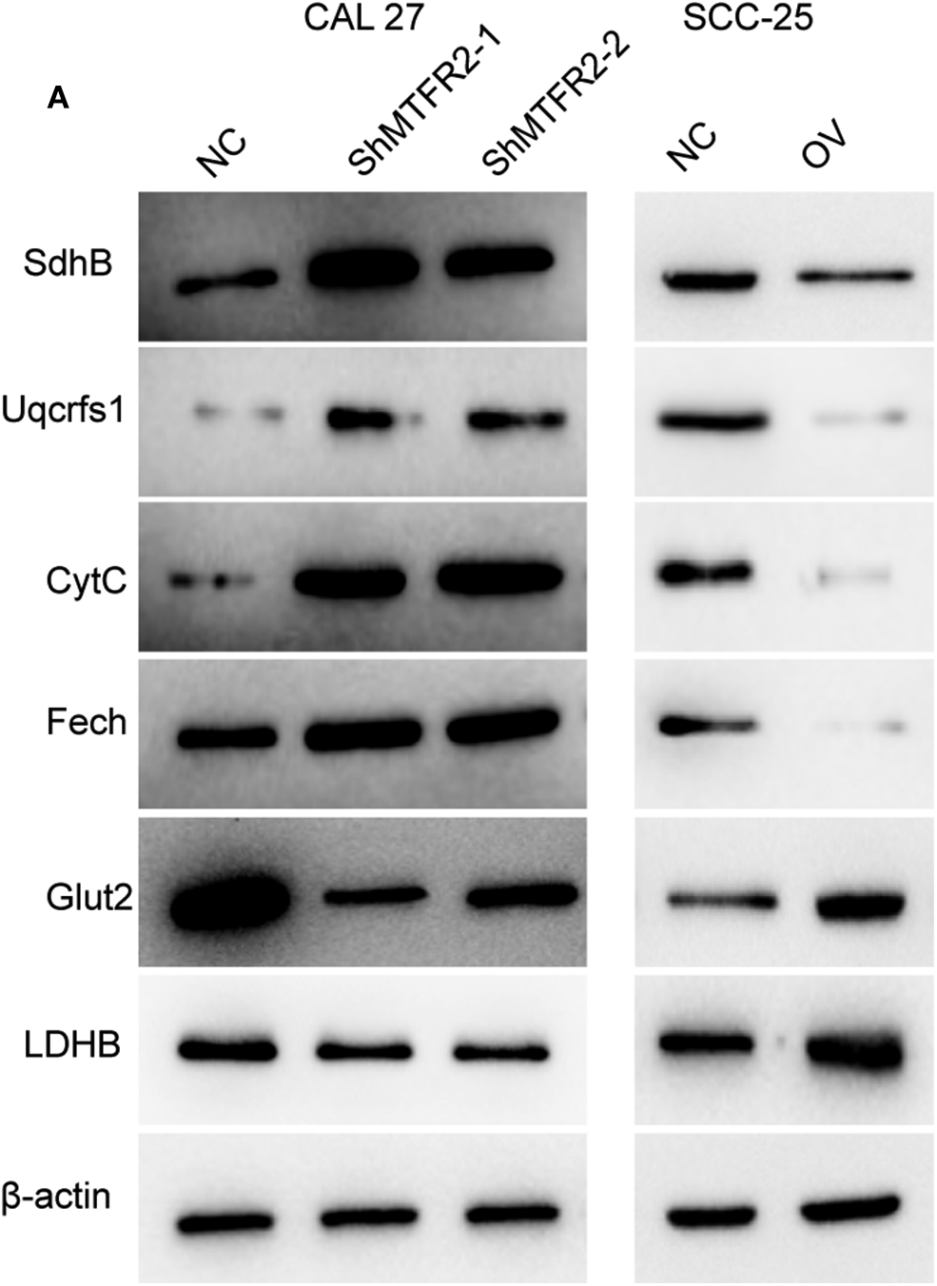
MTFR2 promotes the glycolysis cascade in OSCC. **(A)** western blot of the OXPHOS and glycolysis markers in OSCC.

OXPHOS and glycolysis are complicated reactions in which many enzymes are engaged. To further verify the exact mechanism of MTFR2 in the metabolism of glycose, we next detected the expression pattern of the key enzymes involved in OXPHOS and glycolysis. Enzymes involved in OXPHOS, such as SdhB, Uqcrfs1, CytC and Fech, were downregulated in the MTFR2 knockdown cell line, while the glycolysis enzymes Glut2 and LDHB increased with the overexpression of MTFR2 (Figure 6) (Figure S2A, ^***^p < 0.001). Taken together, we concluded that MTFR2 inhibited the electron transport chain but promoted glycolysis cascades.

## Discussion

OSCC is one of the ten most common cancers worldwide, and the current therapy is mostly surgical operation. Some patients still suffer from metastasis and recurrence. No effective immunotherapy had been applied in clinical practice. Therefore, the identification of more biomarkers and therapeutic targets is needed.

Epithelial-mesenchymal transition (EMT) is a cellular programme cells transform from epithelial status into mesenchymal status (6), this programme was revised by many cytokines. This transformation is critical in physiological program and tumorigenesis in multiple types of cancers. Reports showed that it's crucial in the initiation state of tumorigenesis, among all the subtype of the cancer cells, EMT was reported to be reliable markers reflecting malignant status. When EMT is activated by the cytokines, cells lose cell polarity and epithelial cell junctions such as E-cadherin degraded. The transcription factors promoting the expression of cytoskeletons such as the ZEB family and SNAI family, are activated. Cancer cells degrade the extracellular matrix and developed improved migration and invasion ability. Several cytokines such as TGFβ, IL-6, and EGF was reported to be involved in the activation of EMT (7), such as. Activation of the SMAD family activated by TGFβ triggers EMT (8).

Cancer cells were trended to degrade glucose to produce lactate even in an oxygen-enriched environment, which is known as glycolysis, also known as the Warburg effect (9). There are controversies regarding why glycolysis is predominant in cancer cells (10). Glycolysis produces large amounts of ATP in a short time, but OXPHOS produces more ATP than glycolysis. Glycolysis produces one carbon units for emergency situations; however, lactic acid contains more than one carbon unit and is secreted into the extracellular matrix. The most recognized explanation is that the lactic acid produced by cancer cells helps cancer cells escape the immune system (11).

However, our research showed that MTFR2 promotes the proliferation, migration, invasion and progression of OSCC through regulating glucose metabolism by switching OXPHOS to glycolysis. MTFR2 may be a potential therapeutic target in the future. HIF1α was a well known metabolism regulator, we detect the HIF1α level (Figure S2B, ^***^p < 0.001) and established rescue cell line to re-expression HIF1α in MTFR2 stable knocking down cell line and shHIF1α in SCC-25-OV (Figure S2C, ^***^p< 0.001) and found that the glycolysis totally restored (Figure S2D), indicating that HIF1α switch the metabolism in HIF1α dependent manner.

## Conclusion

In this study, we proved that MTFR2 promotes the proliferation, migration and invasion of oral squamous carcinoma cells through regulating glucose metabolism by switching OXPHOS to glycolysis. MTFR2 may be a potential therapeutic target in the future.

## Data Availability Statement

All datasets generated for this study are included in the article/Supplementary Material.

## Ethics Statement

This study was approved by the Medical Ethics and Human Clinical Trial Committee, and Animal Use and Management Committee at Affliated Hospital of Jiangxi University of Traditional Chinese Medicine. The patients/participants provided their written informed consent to participate in this study.

## Author Contributions

YS designed the research, all authors were engaged into the performance of experiments and data analysis. All authors read and approved the final manuscript.

## Conflict of Interest

The authors declare that the research was conducted in the absence of any commercial or financial relationships that could be construed as a potential conflict of interest.

## Supplementary Material

Figure S1 **(A)** The OCR of NC, OV, and Rescue (OV+ WZB117, 5 μl/ml for 24 h). **(B)** The migration and invasion of the cell lines described above.

Figure S2 **(A)** The relative RNA level of metabolism markers, ^***^*p* < 0.001. **(B)** The relative RNA level and protein level of HIF1α in the cell lines. ^***^*p* < 0.001. **(C)** The relative RNA level and protein level of HIF1α in the cell lines. ^***^*p* < 0.001. **(D)** The OCAR assay in different cell line. **(E)** The ECAR assay in different cell line.

Table S1 The characteristics of patients enrolled.
