# Supplementary material for: MTFR2 Promotes the Proliferation, Migration, and Invasion of Oral Squamous Carcinoma by Switching OXPHOS to Glycolysis
Source: Front Oncol. 2020 May 27;10:858. doi: 10.3389/fonc.2020.00858 (PMC7267185; doi:10.3389/fonc.2020.00858)
Supplement: Figure S1 — (A) The OCR of NC, OV, and Rescue (OV+ WZB117, 5 μl/ml for 24 h). (B) The migration and invasion of the cell lines described above. [file Table_1.pdf]

Characteristics	Patients （n=36）
Age（Years）	
＞60	16
≤60	20
Gender	
Male	21
Female	15
Stage	
I+II	12
III+IV	24
Lymph node	
Negative	25
Positive	11
