# Supplementary figures and images for: MTFR2 Promotes the Proliferation, Migration, and Invasion of Oral Squamous Carcinoma by Switching OXPHOS to Glycolysis
Source: Front Oncol. 2020 May 27;10:858. doi: 10.3389/fonc.2020.00858 (PMC7267185; doi:10.3389/fonc.2020.00858)

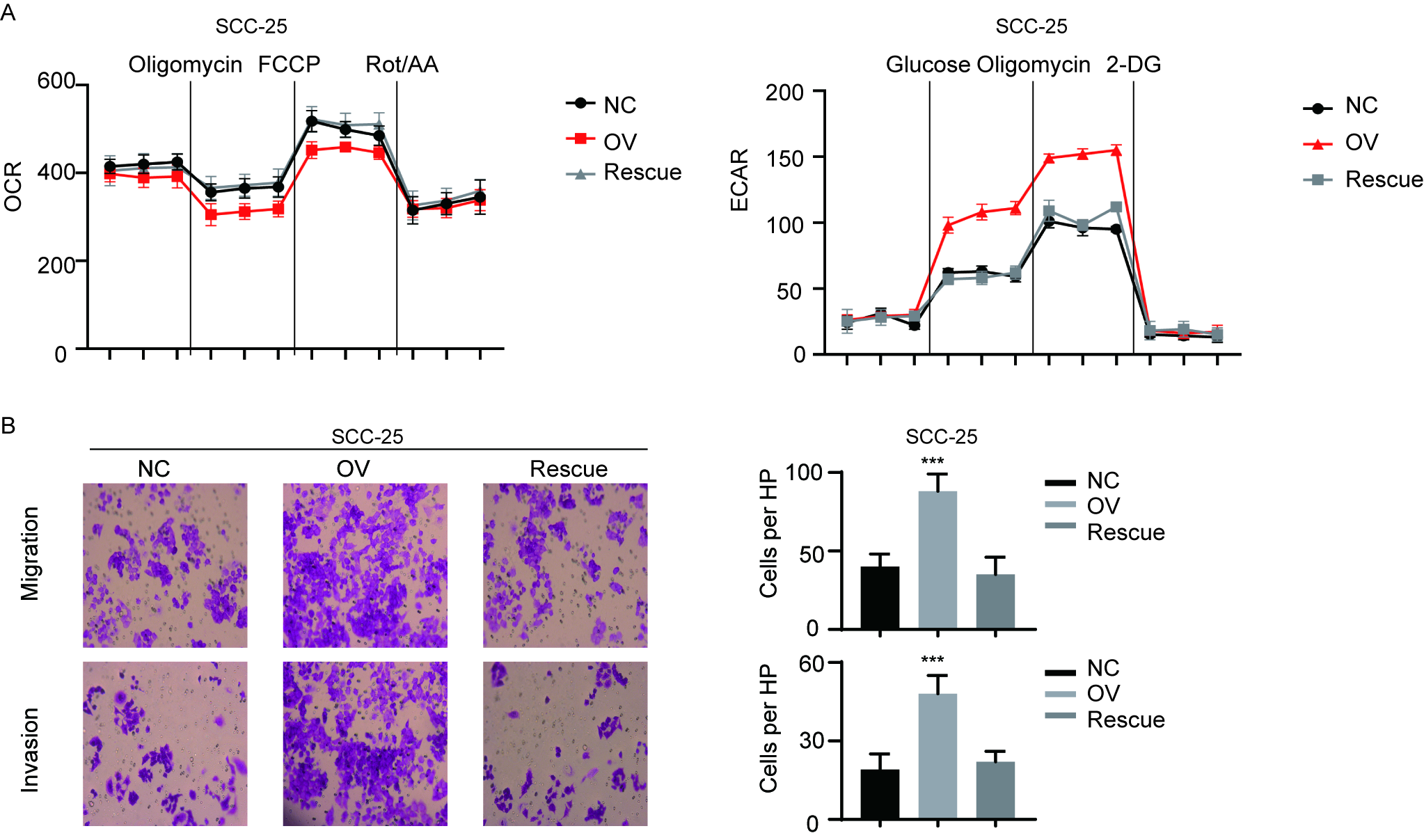

Supplement: Figure S2 — (A) The relative RNA level of metabolism markers, ***p < 0.001. (B) The relative RNA level and protein level of HIF1α in the cell lines. ***p < 0.001. (C) The relative RNA level and protein level of HIF1α in the cell lines. ***p < 0.001. (D) The OCAR assay in different cell line. (E) The ECAR assay in different cell line. [file Image_1.tif]

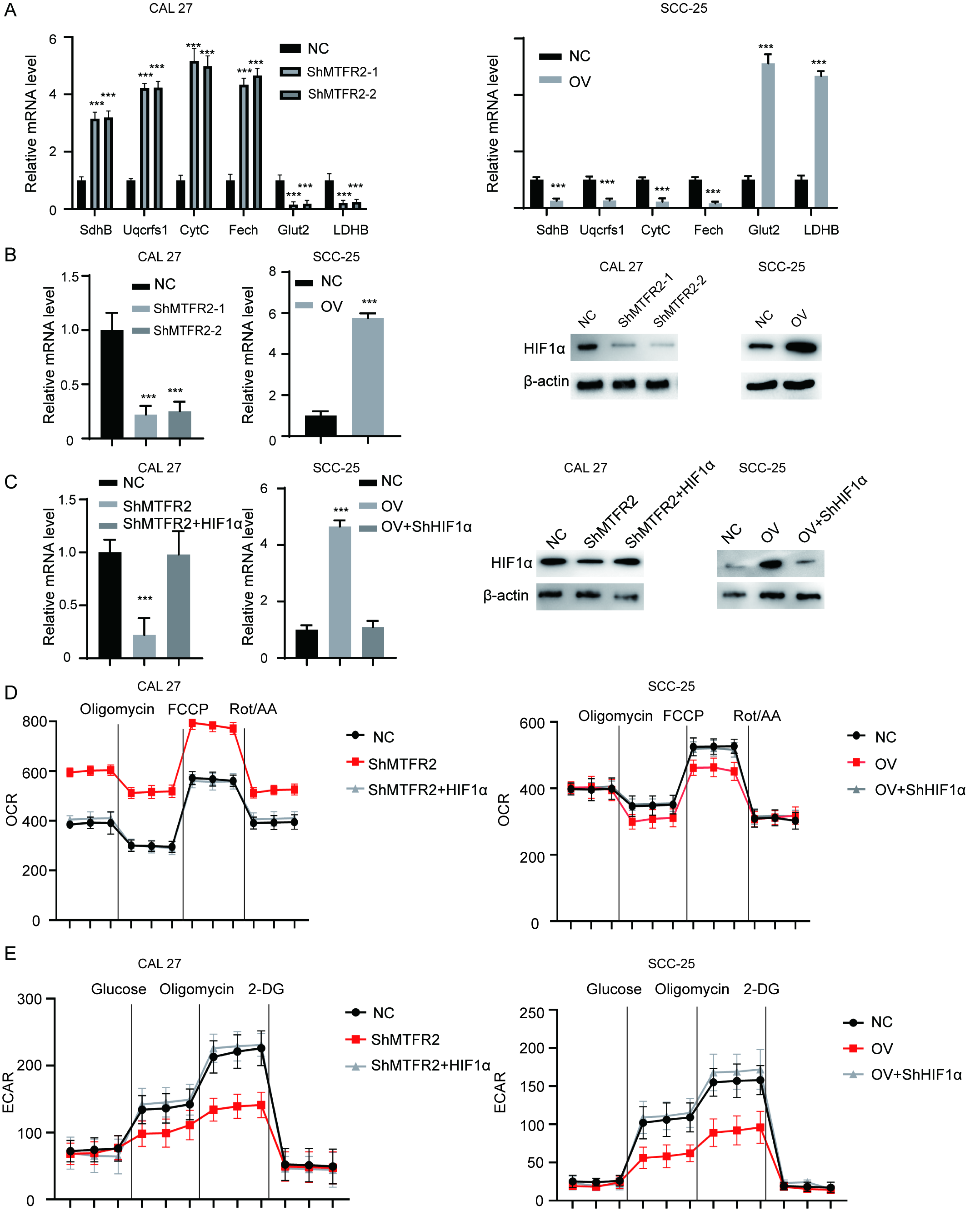

Supplement: Table S1 — The characteristics of patients enrolled. [file Image_2.tif]
